# Synthesis, Characterization, and Antibacterial Studies of Pd(II) and Pt(II) Complexes of Some Diaminopyrimidine Derivatives

**DOI:** 10.1155/2013/549549

**Published:** 2013-03-12

**Authors:** Peter A. Ajibade, Omoruyi G. Idemudia

**Affiliations:** Department of Chemistry, University of Fort Hare, Alice 5700, South Africa

## Abstract

Pd(II) and Pt(II) complexes of trimethoprim and pyrimethamine were synthesized and characterized by elemental analysis, UV-Vis, FTIR, and NMR spectroscopy. The complexes are formulated as four coordinate square planar species containing two molecules of the drugs and two chloride or thiocyanate ions. The coordination of the metal ions to the pyrimidine nitrogen atom of the drugs was confirmed by spectroscopic analyses. The complexes were screened for their antibacterial activities against eight bacterial isolates. They showed varied activities with the active metal complexes showing more enhanced inhibition than either trimethoprim or pyrimethamine. The Pd(II) complexes of pyrimethamine showed unique inhibitory activities against *P. aeruginosa* and *B. pumilus*, and none of the other complexes or the drugs showed any activity against these bacteria isolates. The MIC and MBC determinations revealed that these Pd(II) complexes are the most active. Structure activity relationship showed that Pt(II) complexes containing chloride ions are more active, while for Pd(II) complexes containing thiocyanate ions showed more enhanced activity than those containing chloride ions.

## 1. Introduction

 The discovery of potent group of pyrimidines with pronounced antagonistic effect on folic acid in cultures of *Lactobacilli *[1] led to the development of pyrimethamine and trimethoprim. Pyrimethamine was developed through brilliant feet of organic synthesis guided by biochemical considerations [2]. Additional modifications led to the synthesis of trimethoprim that inhibits bacterial dihydrofolate reductase like other diaminopyrimidines and its consequence selection as antibacterial agent [3–5]. Trimethoprim is a broad-spectrum antimicrobial and also exhibits antiparasitic activities [6]. Due to intensive use and misuse, resistance has emerged against trimethoprim [7].

Development of antimicrobial drugs was hailed as one of the great medical success story of the twentieth century [8]. At present, resistance against antimicrobial agents have become public health problem worldwide [9–15]. In the search for novel drugs against drug resistant diseases, the use of metal complexes has received tremendous attention [16–24] and resulted in a variety of exciting and invaluable drugs such as *cis*-platin [24]. Research are being undertaken in fields such as cancer [25–27], diabetes [28–32], arthritis [33], magnetic resonance imaging [34], metal-mediated antibiotics, antibacterial, antiviral, antiparasitic and radiosensitizers [35–38]. In continuation of our efforts [39–44] to develop metal-based therapeutics agents, the synthesis, characterization, and antibacterial studies of trimethoprim and pyrimethamine are presented.

## 2. Experimental

### 2.1. Materials and Physical Measurements

All reagents and solvents were of analytical grade and used without further purification. Elemental analyses were carried out on a Perkin-Elmer elemental analyzer. Melting point determination was obtained with the Gallenkamp melting point apparatus. Molar conductivity measurement (10^−3^ M solutions in dimethylformamide) was obtained on the CON 6/TDS 6 conductivity/TDS Meter. FTIR spectra of the complexes were recorded as KBr pellets on a Perkin-Elmer paragon 2000 FT-IR spectrophotometer in the range 4000–370 cm^−1^. Electronic spectra of the complexes were recorded on a Perkin-Elmer Lambda 25 spectrophotometer. The ^1^H spectra in DMSO-d_6_ were performed and recorded on a Varian-NMR-vnmr s400 MHz spectrometer at 25°C, using high-power proton decoupling, and pulse sequence: s2pul. Proton chemical shifts in DMSO-d_6_ were referenced to DMSO-d_6_ (^1^H-NMR, *δ*(DMSO) = 2.49 ppm). Chemical shifts for proton resonances are reported relative to tetramethylsilane. PtCl_2_(COD)_2_ and PdCl_2_(CH_3_CN)_2_ were prepared according to literature procedures [45, 46].

### 2.2. Synthesis of Metal Complexes of the Type [M(L)_2_Cl_2_]

A solution containing 1 mmol of the respective metal salts (PtCl_2_(COD)_2_, 0.260 g) and (PdCl_2_(CH_3_CN)_2_, 0.374 g) was added to colorless solutions of trimethoprim (2 mmol, 0.508 g) or pyrimethamine (2 mmol, 0.497 g) in 50 mL of methanol. The mixture was refluxed for 4 h and cooled to room temperature, and the solvent was removed in vacuo. The solid product was dried over CaCl_2_.

### 2.3. Synthesis of Metal Complexes of the Type [M(L)_2_(NCS)_2_]

A solution containing 1 mmol of the respective metal salts (PtCl_2_(COD)_2_, 0.260 g) and (PdCl_2_(CH_3_CN)_2_, 0.374 g) was added to colorless solutions of trimethoprim (2 mmol, 0.508 g) or pyrimethamine (2 mmol, 0.497 g) in 50 mL of methanol. The mixture was refluxed for 1 h, followed by the addition of a colourless solution of NH_4_NCS (2 mmol, 0.152 g) in methanol and refluxed further for 3 h and cooled to room temperature, and the solvent was removed in vacuo. The solid product was dried over CaCl_2_.

### 2.4. Antibacterial Studies

The antimicrobial activity of the synthesized compounds as well as their free ligands was studied by the zone of inhibition technique [47, 48] using *Staphylococcus aureus* (ATCC 6538), *Streptococcus faecalis* (ATCC 29212), *Bacillus cereus* (ATCC 10702), gram(−) *Escherichia coli* (ATCC 8739), *Klebsiella pneumonia* (ATCC 4352), *Proteus vulgaris* (ATCC 6830),* Pseudomonas aeruginosa *(ATCC 19582), and *Bacillus pumilus *(ATCC 14884) typed cultures as obtained from American Type Culture Collection (ATCC). The macrobroth dilution technique [49, 50] was used to determine the MIC. The MIC was taken as the lowest concentration of the tested complexes that shows no visible bacterial growth [51]. Samples of organisms were taken from plates which were used for the MIC test that were with no visible growth and subcultured by way of streaking onto a freshly prepared Mueller Hinton agar medium. MBC was carried out with the method of Olorundare et al. [52] and was taken as the lowest concentration of antibiotic at which all bacteria are killed. These plates were incubated at 35–37°C for 24 h and results taken as the MBC. 

## 3. Results and Discussion

**Figure 1:**
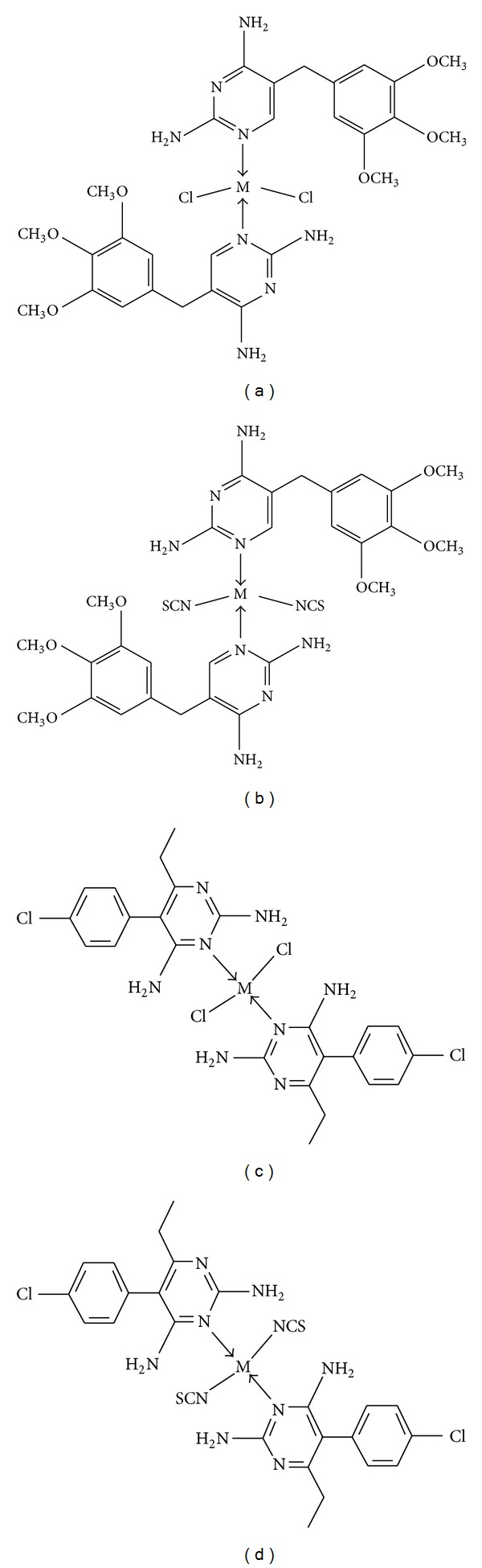
Proposed structures for the metal complexes.

Pd(II) and Pt(II) complexes, trimethoprim and pyrimethamine, have been synthesized and characterized by elemental analysis, UV-Vis, FTIR, and ^1^H and ^13^C-nmr spectroscopy. Conductivity measurements on the complexes showed that they are nonelectrolyte in solution. Generally all the complexes are insoluble both in polar and nonpolar solvents except polar coordinating solvents such as DMSO and DMF. The analytical data for the complexes are presented in Table 1 and proposed structures in Figure 1.

### 3.1. Infrared Spectra

The FTIR spectra of the ligands and metal complexes were compared and assigned on careful comparison. The N—H stretching frequencies of the pyrimidine NH_2_ in the free trimethoprim shifted slightly in the metal complexes. It was observed in the same region, 3332–3461 cm^−1^, as in the free ligands. The slight shift is ascribed to hydrogen bonding and other noncovalent interactions in the metal complexes. The coordination of the metal ions to trimethoprim affected the *v*(C=N) stretching vibrations. The *v*(C=N) that occur at 1635 cm^−1^ in the free trimethoprim ligand shifted to lower frequencies in all the complexes confirming that the metal ions are coordinated directly to the pyrimidine nitrogen atom. Strong vibrations at 2111 and 2120 cm^−1^ in [Pd(tmp)_2_(NCS)_2_] and [Pt(tmp)_2_(NCS)_2_], respectively, are due to *v*(NCS) stretching vibrations and may be attributed to the presence of the thiocyanate ion in the coordination sphere of these complexes [53]. The band observed in the complexes in the region 542−502 cm^−1^ was attributed to *v*(Pd—N) and *v*(Pt—N) [54].

Pyrimethamine possesses four potential coordination sites. A comparison of the spectra of pyrimethamine and the metal complexes showed that the bands due to symmetrical and asymmetrical stretching modes of NH_2_ in the spectrum of pyrimethamine undergo only very slight changes in the complexes. This indicates that the metal ions bond preferentially to pyrimethamine through the nitrogen atom of pyrimidine. The absorption band at 1629 cm^−1^ in the spectrum of the pyrimethamine is attributed to the *v*(C=N) of the pyrimidine ring. It shifted to 1612, 1619, 1639, and 1632 cm^−1^, in [Pd(pyrm)_2_Cl_2_], [Pd(pyrm)_2_(NCS)_2_], [Pt(pyrm)_2_Cl_2_], and [Pt(pyrm)_2_(NCS)_2_], respectively, which is a good indication that pyrimethamine is coordinated to Pd(II) and Pt(II) ions through the N(1) atom of the pyrimidine ring. The appearance of a prominent absorption bands observed at 2112 and 2107 cm^−1^ in the complexes [Pd(pyrm)_2_(NCS)_2_] and [Pt(pyrm)_2_(NCS)_2_], but absent in the free ligand one has is due to *v*(NCS) stretching frequency of the thiocyanate ion [55].

(1) $$\begin{array}{c} \text{M}=\text{Pd}\;\text{or}\;\text{Pt}. \end{array}$$

### 3.2. Electronic Spectra of the Complexes

The effect of complexation on the splitting of the d orbital is more marked for Pd(II) and Pt(II), and consequently their complexes are diamagnetic and majority of them are square planar. The electronic spectra of Pd(II) and Pt(II) complexes like any other square planar complexes can be assigned easily. However, the situation is complicated in the Pt(II) series by the expectation that the d-p transitions will occur at comparable energies to LMCT transitions, and a clear distinction between these two types of transition may be difficult. Pt(II) complexes of trimethoprim do not show any absorption band in the visible region of their electronic spectra, but the Pd(II) complexes display weak absorption bands at around 440 nm which is assigned to  ^1^B_1g_ *←* ^1^A_1g_ and  ^1^A_2g_ *←* ^1^A_1g_  d-d transition of a four coordinate palladium complexes [56]. The absorption band in [Pd(tmp)_2_Cl_2_] is stronger than that of the [Pd(tmp)_2_(NCS)_2_], and this can be attributed to the more intense orange colour of [Pd(tmp)_2_Cl_2_] as compared to that of yellow [Pd(tmp)_2_(NCS)_2_]. The palladium complexes of pyrimethamine show absorption bands at 553 nm, and another absorption band in the region 450–480 nm in both complexes corresponding to  ^1^B_1g_ *←* ^1^A_1g_ and  ^1^A_2g_ *←* ^1^A_1g_ low spin allowed d-d transition [56], respectively. The d-d transition in the platinum complexes is not seen, and this can be evident from its pale yellow colour which makes the MLCT bands dominant, as compared to the deep orange colour of palladium [57]. All the four complexes display high energy absorption band around 300 nm which can be attributed to typical charge transfer transitions in the complexes [56, 57] confirming the square planar geometries proposed for the metal complexes. 

### 3.3. NMR Spectroscopy of the Metal Complexes

^1^H-nmr spectra data of the trimethoprim complexes in d^6^-DMSO shows the presence of some of the proton signals as compared to that of the free trimethoprim ligand [58, 59]. The proton NMR of [Pt(tmp)_2_(NCS)_2_] showed three major peaks at the aromatic region integrating for only three protons at *δ*(ppm) 7.68, 7.46, and 6.61 ppm assigned to (s, 1H, H-4a, *J* = 1.2 Hz), (s, 1H, H-5b), and (s, 1H, H-1b), respectively. The protons of the methyl in the methoxyl group which are equivalent can be observed as a single peak at *δ*(ppm) 1.77 ppm integrated for three protons. In [Pd(tmp)_2_(NCS)_2_] four major peaks can also be observed in the aromatic region but only two of the peaks which are integrated for one proton each was successfully assigned at delta values of 8.25 and 7.96 ppm ascribed to (s, 1H, H-4a) and (s, 1H, H-5b), respectively. The ^1^H-nmr spectrum of [Pt(tmp)_2_Cl_2_] could not be resolved but the ^13^C-nmr gave useful information for the formation of the Pd(II) complex. The coordinations of the two different metals and their contributions in [Pt(tmp)_2_(NCS)_2_] and [Pd(tmp)_2_(NCS)_2_] can be seen from the slight shift of the proton nmr signals assigned in their spectra. 

^1^H and ^13^C-nmr spectra of pyrimethamine [60] were compared to those of the complexes. There was a significant shift in the chemical shift values upon complexation. The ^1^H-nmr spectra of [Pt(pyrm)_2_Cl_2_] in DMSO-d_6_ solutions showed chemical shift at *δ*_(ppm)_ 7.51 (s, 1H, H-5b), 7.26 (s, 1H, H-3b), 6.75 (s, 1H, H-6b), and 6.55 (s, 1H, H-2b). The aliphatic region showed multiple signals between 2.20 and 1.00 ppm which support the presence of an ethyl group of the pyrimethamine ligand, integrating for five protons. In [Pt(pyrm)_2_(NCS)_2_], the chemical shift was observed at *δ*_(ppm)_ 7.53 (s, 2H, H-5b, 3b), 7.28 (s, 1H, H-6b), and 6.98 (s, 1H, H-2b). Once again, the aliphatic region showed multiple signals between 2.20 and 1.00 ppm which support the presence of an ethyl group integrating for five protons. The two amino groups of pyrimethamine exhibit characteristic shifts which can be seen as a broad peak at chemical shift value at around 4.00 ppm in both complexes. 

The ^13^C-nmr spectra of the metal complexes of trimethoprim were compared with that of free trimethoprim ligand [61] with a significant shift in the area that has been affected by the coordination to the metal ions. The signal at 155.91 ppm in the ligand has been shifted upfield to a value of 153.60 ppm in [Pt(tmp)_2_Cl_2_] assigned to C(2a, C6a), both of C=N carbon atom of the pyrimidine ring of trimethoprim. Other ^13^C-nmr signal of trimethoprim were observed at 136.69 ppm assigned to the quaternary carbon C(5a, C5b), 106.59 ppm C(1b, 5b), 60.99 ppm assigned to C7a, the peaks at 56.64 and 32.99 ppm assigned to C(2b, 3b, 4b) and CH_3_ bonded to the oxygen of the methoxyl. The ^13^C-nmr of [Pt(tmp)_2_(NCS)_2_] and [Pd(tmp)_2_(NCS)_2_] both have more peaks than that of the [Pt(tmp)_2_Cl_2_], probably due to the presence of a thiocyanate group NCS in the coordination sphere of these complexes. The resonance at 162.77 and 160.68 ppm in [Pt(tmp)_2_(NCS)_2_] and at 163.10 and 160.51 ppm in [Pd(tmp)_2_(NCS)_2_] corresponding to C=N (C2a, C6a) of the trimethoprim ligand shifted downfield and this can be ascribed to the coordination of the metal to the nitrogen of the pyrimidine ring. The quaternary carbon C5a, C5b chemical shift occurs at 155.94 and 153.53 ppm in [Pt(tmp)_2_(NCS)_2_] and at 154.83 and 153.72 ppm in [Pd(tmp)_2_(NCS)_2_]. The presence of a peak at 135 ppm in both complexes is an indication that the thiocyanate ions are present in the complex and coordinate through the nitrogen. The high intensity peaks at 56.67 and 56.68 ppm in [Pt(tmp)_2_(NCS)_2_] and [Pd(tmp)_2_(NCS)_2_] were assigned to the methoxyl carbon, the peaks at 60.85 ppm in [Pt(tmp)_2_(NCS)_2_] and 60.84 ppm in [Pd(tmp)_2_(NCS)_2_] were assigned to methylene C7a, and lastly the peaks at 33.19 and 33.58 ppm were assigned to the CH_3_ of the aliphatic region.

 The ^13^C-nmr for both platinum complexes of pyrimethamine gave more information in assigning the necessary signals and affirming the probable structure of the complexes. In the spectra of [Pt(pyrm)_2_Cl_2_], the singlet resonance at 163.83 is assigned to carbon atom of C=N of the pyrimidine ring; there was a significant upfield shift which is probably due to the effect of coordination to the Pt(II) ion. The peak at 159.43 ppm was assigned to C5a. The phenyl ring carbon C1b–C6b can be seen in the range of 133.58–107.10 ppm. The aliphatic region consists of two peaks at 26.44 and 13.59 ppm assigned to the methylene and the methyl groups, respectively, from the ethyl of the pyrimethamine ligand. [Pt(pyrm)_2_(NCS)_2_] show a similar trend in the ^13^C-nmr spectrum; the singlet peak at 164.34 ppm was assigned to C(2a) of the pyrimethamine. The thiocyanate carbon is observed at 134.02 ppm [62, 63]. The carbons of the pyrimethamine phenyl ring were found in the range of 133.34–107.65 ppm. The aliphatic region consists of complex peaks at between 29.93 and 25.62 ppm, indicative of the presence of a methylene group. 

### 3.4. Antibacterial Screening of the Metal Complexes

The complexes showed varied antibacterial activities against both gram-positive and gram-negative bacterial isolates (Table 2). The highest zone of inhibition of 34 mm was recorded for [Pd(tmp)_2_Cl_2_] *B*. *cereus*. All Pd and Pt complexes of trimethoprim are active against *E*. *coli *and *P*. *vulgaris*. Their zones of inhibition varied between 28 and 32 mm as against 16 mm shown by trimethoprim drug. Trimethoprim and all its complexes are inactive against *P*. *aeruginosa *and *B*. *pumilus*. Of all the trimethoprim complexes, [Pd(tmp)_2_(NCS)_2_] appear to be the least active, inhibiting only *E*. *coli*, *S*. *faecalis,* and *P*. *vulgaris*. It must also be noted that, it might be least active but it is the only complex of trimethoprim that shows a zone of inhibition of 27 mm against *S*. *faecalis* whereas the other three complexes did not show any visible inhibition. For the trimethoprim complexes, the structure activity relationship showed that both Pd(II) and Pt(II) complexes containing chloride ions are the most active. They are active against two gram positive bacteria and three gram negative bacteria isolates with the Pt complexes, [Pt(tmp)_2_Cl_2_], showing relatively higher zones of inhibition against five bacteria isolates than the Pd(II) complex, [Pd(tmp)_2_Cl_2_]. It must be noted however that high zone of inhibition may not reveal for certain that a particular compound is a stronger antibacterial agent since this may be attributed to factors such as the rate of diffusion of the antibacterial agents and the amount of bacterial isolates present in a certain amount of agar solution [64].

The inhibition of the bacteria isolates by the pyrimethamine complexes are relatively fewer than those of complexes of trimethoprim (Table 2). However, the results showed that Pd(II) complexes of pyrimethamine, [Pd(pyrm)_2_Cl_2_] and [Pd(pyrm)_2_(NCS)_2_], have zones of inhibition of 30 and 33 mm against *P*. *aeruginosa* and 14 and 33 mm against *B*. *pumilus*. None of the complexes of trimethoprim and neither trimethoprim nor pyrimethamine showed any visible activity against these bacteria isolates. The highest zones of inhibition of 33 mm were observed for [Pd(pyrm)_2_(NCS)_2_] against *P*. *aeruginosa* and *K*. *pneumonia*. Pyrimethamine inhibited the growth of six bacteria isolates whereas three of its metal complexes inhibited four bacteria isolates while [Pt(pyrm)_2_Cl_2_] inhibited only three bacteria isolates. In the trimethoprim metal complexes, the complexes containing the chloride ions are generally showed better antibacterial activities than those with NCS ions. In the pyrimethamine metal complexes, the NCS ion is the anion of choice for enhanced activity. The minimum inhibition concentrations (MICs) and minimum bactericidal concentrations (MBCs) of the compounds were evaluated and presented in Tables 3 and 4, respectively. It shows that the Pd(II) complexes of pyrimethamine are more active than the Pt(II) complexes of either trimethoprim or pyrimethamine. The lowest MIC values of 0.31 mg/mL were recorded for the Pd complexes: [Pd(pyrm)_2_Cl_2_] against *K*. *pneumonia *and [Pd(pyrm)_2_(NCS)_2_] against *S*. *aureus*. The complexes also have MIC values of 0.63 mg/mL against *S*. *aureus* and *B*. *pumilus* for [Pd(pyrm)_2_Cl_2_] while [Pd(pyrms)_2_(NCS)_2_] values are against *B*. *pumilus *and *K*. *pneumonia*. Their MBC values against these bacteria isolates are also the lowest indicating their high antibacterial activities.

## 4. Conclusions

We report the synthesis, characterization, and antibacterial studies of Pd(II) and Pt(II) complexes of trimethoprim and pyrimethamine. The complexes formulated as four coordinate square planar species consisting of two molecules of either trimethoprim or pyrimethamine and two chloride or thiocyanate ions. The complexes were characterized by elemental analysis, electronic, FTIR, and NMR spectroscopy. Spectroscopic analyses confirmed the coordination of the metal ions to the drug through the pyrimidine nitrogen atom. The antibacterial screening of the complexes showed varied activities but they are more active than the drugs. The MIC and MBC determinations revealed that the Pd(II) metal complexes are the most active.

## Figures and Tables

**Table 1:** Analytical data and some physical properties of the metal complexes.

			Analytical Data (%)					
Complexes	Molecular formulae	Colour	C	H	N	Yield (%)	M.P (°C)	Cond. *µ*S
			Found (calc.)	Found (calc.)	Found (calc.)			
[Pd(tmp)_2_Cl_2_]	C_28_H_36_N_8_Cl_2_O_6_Pd	Orange	43.94 (44.37)	5.13 (4.79)	15.47 (14.78)	91	248–250	11.42
[Pd(tmp)_2_(SCN)_2_]	C_30_H_36_N_10_O_6_S_2_Pd	Yellow	44.42 (44.86)	3.97 (4.52)	17.01 (17.44)	92	242–245	9.03
[Pt(tmp)_2_Cl_2_]	C_28_H_26_N_8_Cl_2_O_6_Pt	Pale yellow	40.10 (39.72)	3.79 (4.29)	13.72 (13.24)	92	135–137	10.13
[Pt(tmp)_2_(SCN)_2_]	C_30_H_36_N_10_O_6_S_2_Pt	Pale yellow	40.94 (40.40)	4.22 (4.07)	16.27 (15.70)	88	167–171	7.02
[Pd(pyrm)_2_Cl_2_]	C_24_H_26_N_8_Cl_4_Pd	Orange	43.65 (42.72)	4.26 (3.88)	16.69 (16.61)	90	259–261	15.62
[Pd(pyrm)_2_(SCN)_2_]	C_26_H_26_N_10_Cl_2_S_2_Pd	Orange	43.46 (43.37)	3.76 (3.64)	19.66 (19.45)	92	80–82	40.80
[Pt(pyrm)_2_Cl_2_]	C_24_H_26_N_8_Cl_4_Pt	Pale yellow	37.99 (37.76)	3.88 (3.43)	14.93 (14.68)	89	200–202	39.80
[Pt(pyrm)_2_(NCS)_2_]	C_26_H_26_N_10_Cl_2_S_2_Pt	Pale yellow	39.90 (38.62)	3.77 (3.24)	16.98 (17.32)	97	180-181	52.80

**Table 2:** Zone of inhibition exhibited by metal complexes at 40 mg/mL (mm).

Complexes	*E. coli *	*P. aeruginosa *	*S. aureus *	*S. faecalis *	*B. cereus *	*B. pumilus *	*K. pneumonia *	*P. vulgaris *
[Pd(tmp)_2_Cl_2_]	30.0	NI	29.0	NI	32.0	NI	26.0	32.0
[Pd(tmp)_2_(NCS)_2_]	28.0	NI	NI	27.0	NI	NI	NI	28.0
[Pt(tmp)_2_Cl_2_]	32.0	NI	32.0	NI	34.0	NI	30.0	28.0
[Pt(tmp)_2_(NCS)_2_]	30.0	NI	25.0	NI	32.0	NI	NI	30.0
[Pd(pyrm)_2_Cl_2_]	NI	30	12	NI	NI	21	14	NI
[Pd(pyrm)_2_(NCS)_2_]	NI	33	29	NI	NI	20	33	NI
[Pt(pyrm)_2_Cl_2_]	14	NI	20	26	NI	NI	NI	NI
[Pt(pyrm)_2_(NCS)_2_]	20	NI	18	NI	28	NI	NI	30
Pyrimethamine	11	NA	13	12	10	NA	15	12
Trimethoprim	15.0	NA	12.0	18.0	17.0	NA	20.0	16.0

NI: no inhibition; NA: not applicable.

**Table 3:** MIC values (mg/mL) of the metal complexes on selected bacteria.

Complexes	*E. coli *	*P. aeruginosa *	*S. aureus *	*S. faecalis *	*B. cereus *	*B. pumilus *	*K. pneumonia *	*P. vulgaris *
[Pd(pyrm)_2_Cl_2_]	NI	5.0	0.63	NI	NI	0.63	0.31	NI
[Pd(pyrm)_2_(NCS)_2_]	NI	5.0	0.31	NI	NI	0.63	0.63	NI
[Pt(pyrm)_2_Cl_2_]	20.0	NI	10.0	5.0	NI	NI	NI	NI
[Pt(pyrm)_2_(NCS)_2_]	10.0	NI	10.0	NI	5.0	NI	NI	5.0
[Pd(tmp)_2_Cl_2_]	20.0	NI	10.0	NI	5.0	NI	5.0	10.0
[Pd(tmp)_2_(NCS)_2_]	10.0	NI	NI	10.0	NI	NI	NI	NI
[Pt(tmp)_2_Cl_2_]	20.0	NI	10.0	NI	10.0	NI	5.0	10.0
[Pt(tmp)_2_(NCS)_2_]	20.0	NI	10.0	NI	10.0	NI	NI	5.0
Pyrimethamine	20.0	NA	20.0	20.0	10.0	NA	20.0	10.0
Trimethoprim	20.0	NA	10.0	20.0	10.0	NA	10.0	10.0

**Table 4:** MBC values (mg/mL) of the metal complexes on selected bacteria.

Complexes	*E. coli *	*P. aeruginosa *	*S. aureus *	*S. faecalis *	*B. cereus *	*B. pumilus *	*K. pneumonia *	*P. vulgaris *
[Pd(tmp)_2_Cl_2_]	>20.0	NI	20.0	NI	5.0	NI	10.0	20.0
[Pd(tmp)_2_(NCS)_2_]	10.0	NI	NI	>20.0	NI	NI	NI	NI
[Pt(tmp)_2_Cl_2_]	20.0	NI	>20.0	NI	10.0	NI	10.0	>20.0
[Pt(tmp)_2_(NCS)_2_]	>20.0	NI	10.0	NI	>20.0	NI	NI	20.0
[Pd(pyrm)_2_Cl_2_]	NI	10.0	2.5	NI	NI	2.5	0.31	NI
[Pd(pyrm)_2_(NCS)_2_]	NI	10.0	0.63	NI	NI	1.25	2.50	NI
[Pt(pyrm)_2_Cl_2_]	20.0	NI	10.0	10.0	NI	NI	NI	NI
[Pt(pyrm)_2_(NCS)_2_]	>20.0	NI	20.0	NI	10.0	NI	NI	20.0
Pyrimethamine	>20.0	NA	>20.0	>20.0	>20.0	NA	20.0	>20.0
Trimethoprim	>20.0	NA	20.0	>20.0	20.0	NA	20.0	20.0

## References

[1] Falco EA, Goodwin LG, Hitchings GH, Rollo IM, Russel PB (1951). 2,4-diaminopyrimidines- a new series of antimalarials. *British Journal of Pharmacology and Chemotherapy*.

[2] Roth B, Fmco EA, Hitchings GH, Bushby SRM (1962). 5-Benzyl-2, 1-diaminopyrimidines as antibacterial agents. I. Synthesis and antibacterial activity in vitro. *Journal of Medicinal and Pharmaceutical Chemistry*.

[3] Hitchings GH, Smith SL (1980). Dihydrofolate reductases as targets for inhibitors. *Advances in Enzyme Regulation*.

[4] Falco EA, Goodwin LG, Hitchings GA, Rollo IM, Russel PB (1951). 2:4-diaminopyrimidines- a new series of antimalarials. *British Journal of Pharmacology and Chemotherapy*.

[5] Hitchings GH (1969). Species differences among dihydrofolate reductases as a basis for chemotherapy. *Postgraduate Medical Journal*.

[6] Burchall JJ, Corcoran JW, Hahn FE (1975). *Antibiotics*.

[7] Then RL (1993). History and future of antimicrobial 2, 4-diaminopyrimidines. *Journal of Chemotherapy*.

[8] Cook BM, Mohandas N, Copel RL (2004). Malaria and the red blood cell membrane. *Seminars in Hematology*.

[9] Chu DTW, Plattner JJ, Katz L (1996). New directions in antibacterial research. *Journal of Medicinal Chemistry*.

[10] Verhoef J, Fluit A (2006). Surveillance uncovers the smoking gun for resistance emergence. *Biochemical Pharmacology*.

[11] Leeb M (2004). Antibiotics: a short in the arm. *Nature*.

[12] Ajibade PA, Zulu NH (2010). Synthesis, characterization, and antibacterial activity of metal complexes of phenylthiourea: the X-ray single crystal structure of [Zn(SC(NH_2_)NHC_6_H_5_)_2_(OOCCH_3_)_2_] C_2_H_5_OH. *Journal of Coordination Chemistry*.

[13] Loginova NV, Koval’chuk TV, Zheldakova RA (2006). Synthesis and biological evaluation of copper (II) complexes of sterically hindered o-aminophenol derivatives as antimicrobial agents. *Bioorganic and Medicinal Chemistry Letters*.

[14] World Health Organization (2004). *The World Health Report 2004-Changing History, Annex Table 2: Deaths By Cause, Sex and Mortality Stratum in WHO Regions, Estimates For 2002*.

[15] Namba K, Zheng X, Motoshima K (2008). Design and synthesis of benzenesulfananilides active against methicillin resistant staphylococcus aureus and vancomycin-resistant enterococcus. *Bioorganic and Medicinal Chemistry Letters*.

[16] Rajapakse CSK, Martinez A, Naoulou B (2009). Synthesis, characterization, and in vitro antimalarial and antitumor activity of new ruthenium(II) complexes of chloroquine. *Inorganic Chemistry*.

[17] Wee HA, Daldini E, Scolaro C, Scopelliti R, Juillerat-Jeannerat L, Dyson PJ (2006). Development of organometallic ruthenium-arene anticancer drugs that resist hydrolysis. *Inorganic Chemistry*.

[18] Allardyce CS, Dorcier A, Scolaro C, Dyson PJ (2005). Development of organometallic (organo-transition metal) pharmaceuticals. *Applied Organometallic Chemistry*.

[19] Refat MS, El-Shazly SA (2010). Identification of a new anti-diabetic agent by combining VOSO4 and vitamin E in a single molecule: studies on its spectral, thermal and pharmacological properties. *European Journal of Medicinal Chemistry*.

[20] Hussein BHM, Hassan AA, Mona FEA, Abdullah I EF (2012). A novel anti-tumor agent, Ln(III) 2-thioacetate benzothiazole induces anti-angiogenic effect and cell death in cancer cell lines. *European Journal of Medicinal Chemistry*.

[21] Thompson KH, Chiles J, Yuen VG, Tse J, McNeill JH, Orvig C (2004). Comparison of anti-hyperglycemic effect amongst vanadium, molybdenum and other metal maltol complexes. *Journal of Inorganic Biochemistry*.

[22] Brichard SM, Henquin J-C (1995). The role of vanadium in the management of diabetes. *Trends in Pharmacological Sciences*.

[23] Sekhon BS, Gandhi L (2006). Medicinal uses of inorganic compounds-1. *Resonanceno*.

[24] Lebwohl D, Canetta R (1998). Clinical development of platinum complexes in cancer therapy: an historical perspective and an update. *European Journal of Cancer*.

[25] Hartinger CG, Jakupec MA, Zorbas-Seifried S (2008). KP1019, a new redox-active anticancer agent-preclinical development and results of a clinical phase I study in tumor patients. *Chemistry and Biodiversity*.

[26] Sava G, Bergamoa A, Dyson PJ (2011). Metal-based antitumour drugs in the post-genomic era: what comes next?. *Dalton Transactions*.

[27] Sun RWY, Ma DL, Wong ELM, Che CM (2007). Some uses of transition metal complexes as anti-cancer and anti-HIV agents. *Dalton Transactions*.

[28] Sakurai H, Kojima Y, Yoshikawa Y, Kawabe K, Yasui H (2002). Antidiabetic vanadium(IV) and zinc(II) complexes. *Coordination Chemistry Reviews*.

[29] Kiss T, Jakusch T, Hollender D (2008). Biospeciation of antidiabetic VO, (IV) complexes. *Coordination Chemistry Review*.

[30] Sekar N, Li J, Shechter Y (1996). Vanadium salts as insulin substitutes: mechanisms of action, a scientific and therapeutic tool in diabetes mellitus research. *Critical Reviews in Biochemistry and Molecular Biology*.

[31] Thompson KH, Orvig C (2006). Vanadium in diabetes: 100 years from Phase 0 to Phase I. *Journal of Inorganic Biochemistry*.

[32] Thompson KH, McNeill JH, Orvig C (1999). Vanadium compounds as insulin mimics. *Chemical Reviews*.

[33] Messori L, Marcon G (2004). Gold complexes in the treatment of rheumatoid arthritis. *Metal Ions Biological Systems*.

[34] Caravan P, Ellison JJ, McMurry TJ, Lauffer RB (1999). Gadolinium(III) chelates as MRI contrast agents: structure, dynamics, and applications. *Chemical Reviews*.

[35] Garoufis A, Hadjikakou SK, Hadjiliadis N (2009). Palladium coordination compounds as anti-viral, anti-fungal, anti-microbial and anti-tumor agents. *Coordination Chemistry Reviews*.

[36] Orvig C, Abrams MJ (1999). Medicinal inorganic chemistry. *Chemical Review*.

[37] Guo Z, Sadler PJ (1999). Metals in medicine. *Angew Chemie International Edition*.

[38] Abrams MJ, Murrer BA (1993). Metal compounds in therapy and diagnosis. *Science*.

[39] Ajibade PA, Kolawole GA, O’Brien P (2007). Metal complexes of 4-amino-N-(2-pyrimidinyl)benzene sulfonamide: synthesis, characterization and antiprotozoal studies. *Synthesis and Reactivity in Inorganic, Metal-Organic and Nano-Metal Chemistry*.

[40] Ajibade PA, Kolawole GA (2008). Synthesis, characterization, antiplasmodial and antitrypanosomal activity of some metal(III) complexes of sulfadiazine. *Bulletin of the Chemical Society of Ethiopia*.

[41] Ajibade PA, Kolawole GA (2008). Synthesis, characterization and antiprotozoal studies of some metal complexes of antimalarial drugs. *Transition Metal Chemistry*.

[42] Ajibade PA, Kolawole GA (2008). Synthesis, characterization and in vitro antiprotozoal studies of iron(III) complexes of some antimalarial drugs. *Journal of Coordination Chemistry*.

[43] Ajibade PA (2008). Metal complexes in the management of parasitic diseases: in vitro antiprotozoal studies of metal complexes of some antimalarial drugs. *Current Science*.

[44] Ajibade PA, Kolawole GA (2010). Cobalt(III) complexes of some antimalarial drugs: synthesis, characterization, and in vitro antiprotozoal studies. *Synthesis and Reactivity in Inorganic, Metal-Organic and Nano-Metal Chemistry*.

[45] Anderson GK, Lehn M (1990). Bis(benzonitrile)dichloro complexes of palladium and platinum. *Inorganic Synthesis*.

[46] Manav N, Mishra AK, Kaushik NK (2004). Triphenyl phosphine adducts of platinum(IV) and palladium(II) dithiocarbamates complexes: a spectral and in vitro study. *Spectrochimica Acta A*.

[47] Grierson DS, Afolayan AJ (1999). Antibacterial activity of some indigenous plants used for the treatment of wounds in the Eastern Cape, South Africa. *Journal of Ethnopharmacology*.

[48] Russell AD, Furr JR (1977). The antibacterial activity of a new chloroxylenol preparation containing ethylenediamine tetraacetic acid. *Journal of Applied Bacteriology*.

[49] Ibrahim MB, Owonubi MO, Onaolapo JA (1997). Antimicrobial effects of extracts of leaf, stem, and root-bark of Anogiessus leicarpus on Staphylococcus aureus NCTC, 8190, Escherichia coli NCTC, 10418 and Proteus vulgaris NCTC, 4636. *Journal of Pharmaceutical Research and Development*.

[50] Akinpelu DA, Kolawole DO (2004). Phytochemistry and antimicrobial activity of leaf extractof Piliostigma thonningii (Schum). *Science Focus*.

[51] Nishizawa K, Hirano M, Kimura A (1998). Evaluation of the antimicrobial activity of carbapenem and cephem antibiotics against Pseudomonas aeruginosa isolated from hospitalized patients. *Journal of Infection and Chemotherapy*.

[52] Olorundare EE, Emudianughe TS, Khasar GS, Koteyi SA, Irobi N (1992). Antibacterial properties of leave extract of Cassia alata. *Biological Research*.

[53] Shaker SA, Farina Y, Mahmmod S, Eskender M (2009). Preparation and study of mixed ligand complexes of caffeine and cyanate with some metal ions. *Australian Journal of Basic and Applied Sciences*.

[54] Liu J, Zhang B, Wu B, Zhang K, Hu S (2007). The direct electrochemical synthesis of Ti(II), Fe(II), Cd(II), Sn(II), and Pb(II) cwith N, N-bis(Salicylidene)-o-phenylenediamine. *Turkish Journal of Chemistry*.

[55] Shaker SA, Farina Y (2009). Preparing and characterization of some mixed ligand Complexes of 1, 3, 7-Trimethylxanthin, *γ*-Picoline and thiocyanate with some metal ions. *American Journal of Scientific Research*.

[56] Charlson AJ, McArdle NT, Watton EC (1981). The induction of filamentous growth in Escherichia Coli by a palladium(II) complex of L-serine. *Inorganica Chimica Acta C*.

[57] Grant GJ, Goforth AM, van Derver DG, Pennigton WT (2004). Homoleptic platinum(II) and palladium(II) complexes of 1,5,9-trithiacyclododecane: the crystal structures of [Pt(12S3)_2_](PF_6_)_2_
*·* 2CH_3_NO_2_ and [Pd(12S3)_2_](BF_4_)_2_
*·* 0.5H_2_O. *Inorganic Chimica Acta*.

[58] Koetzle TF, Williams GJB (1976). The crystal and molecular structure of the antifolate drug trimethoprim (2,4-diamino-5-(3,4,5-trimethoxybenzyl)pyrimidine). A neutron diffraction study. *Journal of American Chemical Society*.

[59] Bergh JJ, Breytenbach JC, Wessels PL (1989). Degradation of trimethoprim. *Journal of Pharmaceutical Sciences*.

[60] de Araújo MVG, Vieira EKB, Lázaro GS (2007). Inclusion complexes of pyrimethamine in 2-hydroxypropyl-*β*-cyclodextrin: characterization, phase solubility and molecular modelling. *Bioorganic and Medicinal Chemistry*.

[61] Simo B, Porello L, Ortiz R, Castineiras A, Latorre J, Canton E (2000). Interactions of metal ions with a 2, 4-diaminopyrimidine derivative (trimethoprim): antimicrobial studies. *Journal of Inorganic Biochemistry*.

[62] Nazeeruddin MK, Zakeeruddin SM, Humphry-Baker R, Gorelsky SI, Lever ABP, Grätzel M (2000). Synthesis, spectroscopic and a ZINDO study of cis- and trans-(X2)bis(4,4′-dicarboxylic acid-2,2′-bipyridine)ruthenium(II) complexes (X = Cl-, H2O, NCS-). *Coordination Chemistry Reviews*.

[63] Li X, Gui J, Yang H, Wu W, Li F, Huang HC (2008). A new carbozole-based phenanthrenyl ruthenium complex as sensitizer for a dye-sensitized solar cell. *Inorganic Chimica Acta*.

[64] Rios JL, Recio MC, Villar A (1988). Screening methods for natural products with antimicrobial activity: a review of the literature. *Journal of Ethnopharmacology*.

